# Usefulness of Arterial Stiffness as an Integrated Marker of Cardiovascular Risk

**DOI:** 10.1111/jch.70038

**Published:** 2025-03-24

**Authors:** Grethzel Prado, María J. Forner, Oscar Calaforra, Sara Vela, Carlos Bea, Gernot Pichler, Ana de Gracia, Lucas Serna, Enrique Rodilla, Josep Redon, Fernando Martínez‐García

**Affiliations:** ^1^ Emergency Department La Fe University and Polytechnic Hospital Valencia Spain; ^2^ Internal Medicine Department Hypertension Unit Clinical Hospital of Valencia Valencia Spain; ^3^ Cardiometabolic Research Group Research Institute of the Clinical Hospital of Valencia (INCLIVA) Valencia Spain; ^4^ University of Valencia Valencia Spain; ^5^ Research Institute of the Clinical Hospital La Fe Valencia Spain; ^6^ Karl Landsteiner Institute for Cardiovascular and Critical Care Research Vienna Austria; ^7^ Department of Cardiology Clinic Floridsdorf Vienna Austria; ^8^ Internal Medicine Department, Hypertension and Vascular Risk Unit Sagunto University Hospital Sagunto Spain; ^9^ Department of Medicine, Universidad Cardenal Herrera‐CEU CEU Universities Valencia Spain

**Keywords:** complications, hypertension (HTN), longitudinal studies, pulse wave analysis, vascular stiffness (AS)

## Abstract

We analyzed the usefulness of the carotid‐femoral pulse wave velocity (cfPWV) as an integrated marker for hypertension (HTN)‐mediated organ damage (HMOD) and cardiovascular (CV) risk in a cohort with repeated measurements. A total of 1031 patients, 80% of whom had HTN, underwent cfPWV determinations by SphygmoCor. An HMOD score was developed, including microalbuminuria, left ventricular hypertrophy (LVH), intima‐media thickness (IMT), and carotid plaques. CV complications included atrial fibrillation (AF), heart failure (HF), stroke, ischemic heart disease (IHD), peripheral artery disease (PAD), or CV death. Survival curves based on Cox regression adjusted for age and systolic blood pressure (SBP), along with Harrell's *C* statistic, were assessed. There was a trend toward higher cfPWV across categories of the HMOD score. Significant correlations were found among different AS parameters and blood pressure (BP) levels. Age and SBP were highly correlated with cfPWV. Among the 174 patients with at least two cfPWV measurements, there were 12 CV complications over a follow‐up period of 2.4 years. The first and second cfPWV measurements, as well as the delta values, were significantly higher in those with CV complications, with most patients experiencing an increase in PWV during follow‐up of ≥ 1 m/s. Survival curves significantly differed among tertiles of PWV and the delta, particularly for the second PWV determination, which also showed the highest predictive value (Harrell's *C* = 0.86). The optimal threshold to predict complications was 9.10 m/s. Our findings suggest that cfPWV represents a promising integrated marker of HMOD, potentially serving as a surrogate endpoint for CV risk.

## 1 Introduction

Cardiovascular diseases (CVD) are the leading cause of death globally, and hypertension (HTN) is the primary risk factor [1]. The prevalence of HTN in Europe is 30%–45% and is expected to rise [2]. Identifying individuals with high cardiovascular (CV) risk for intensive treatment is crucial, although many CV events occur in those with low or intermediate risk [3]. Therefore, detection of subclinical HTN‐mediated organ damage (HMOD) is vital [4].

Non‐invasive techniques, such as carotid‐femoral pulse wave velocity (cfPWV), are essential in predicting CV risk, as they are independent markers of CV events and mortality [5]. Factors influencing arterial stiffness (AS) include age, blood pressure (BP), and, to a lesser degree, sex and classic CV risk factors [6]. Antihypertensive drugs, particularly renin‐angiotensin system (RAS) blockers, reduce AS more effectively by remodeling small arteries [7]. Other CV drugs such as statins, iSGLT2 inhibitors, and GLP‐1 agonists may also reduce AS by lowering oxidative stress [8, 9, 10, 11]. Despite the potential of cfPWV as a surrogate marker for CV events, the SPARTE trial showed no benefits in guiding treatment using PWV over standard treatments [12].

Although great progress has been made in establishing the relationship between AS and CV risk, little is known about intra‐patient variability and the clinical utility of serial AS measurements in practice. Moreover, while a fully validated composite score that integrates both dichotomous and quantitative measures of HMOD is not yet established, several studies underscore the need for such an approach. For instance, recent studies have demonstrated that the cumulative presence of HMOD in multiple sites is an independent risk factor for CV events not only in HTN but also in the community [13, 14]. In this context, PWV has emerged as a practical and non‐invasive surrogate marker of AS that correlates well with the cumulative vascular injury induced by sustained high BP [15]. Collectively, these findings highlight the need for a composite score that accurately reflects the magnitude of organ damage in HTN across multiple sites, suggesting that PWV could be a simple and effective way to capture this risk in an integrated manner.

This study aims to develop a composite organ damage score and evaluate whether cfPWV is a simple and effective method for assessing organ damage. Additionally, the correlation between cfPWV and other AS measurements, along with the potential clinical value of cfPWV as a surrogate endpoint for CV risk, will be evaluated.

## 2 Methods

### 2.1 Study Design

This was a retrospective observational study based on data from a cohort of 1031 patients who were followed up in a tertiary hospital's HTN unit from January 2009 to April 2018 and had at least one cfPWV determination.

### 2.2 Study Population

The selected population consisted of adults who were referred to our HTN unit because of diagnosed or suspected HTN or other CV risk factors. All patients underwent a complete anamnesis, including sex, age, smoking, alcohol use, illicit drug use, personal or familial history of other CV risk factors, including type 2 diabetes or dyslipidemia, and previous CV events.

All patients underwent complete physical examination, and most were selected for extensive CV phenotyping. The majority of subjects (*n* = 857) had only one cfPWV, but 174 had two cfPWVs with an average follow‐up period between the two measurements of 2.4 years.

### 2.3 Procedures

#### 2.3.1 Anthropometrics

Weight was measured on an Asimed Barys Plus scale with patients barefoot and wearing light clothing. Height was measured using a stadiometer. Body mass index (BMI) was calculated as weight (kg)/height (m^2^). Waist circumference was measured at the midpoint between the iliac crest and the bottom of the ribs using a flexible tape.

#### 2.3.2 BP Measurement

BP was measured using a validated Omron M6 Comfort device with an appropriate cuff on the nondominant arm. Three readings were taken 1–2 min apart in a quiet room, with the patient seated and the arm supported at heart level. The average of the last two readings was used as the true BP value. Additional measurements were taken if discrepancies greater than 10 mmHg in systolic blood pressure (SBP) or arrhythmias were noted.

#### 2.3.3 Ambulatory Blood Pressure Monitoring (ABPM)

BP was recorded every 20 min during the day and night using Spacelabs 90202, 90207, and 90217 monitors. The data were considered valid if less than 30% of the readings were missing, with a minimum of 20 readings during the day and seven at night. BP was analyzed for daytime (7:00 a.m. to 11:00 p.m.), nighttime (11:00 p.m. to 7:00 a.m.), and 24‐h periods.

Pulse wave analysis (PWA) and cfPWV with the SphygmoCor device (SphygmoCor; AtCor Medical): PWA was performed using applanation tonometry of the radial artery with the patient seated. SBP, diastolic blood pressure (DBP), MAP, and augmentation pressure (AP) were recorded at brachial and central (aortic) levels. Augmentation Index (AIx) was calculated as AIx = AP/PP × 100 and corrected for HR at 75 bpm. The cfPWV was measured between the right carotid and femoral arteries. The length of the arterial segment was estimated by subtracting the distance from the carotid location to the suprasternal notch from the distance between the suprasternal notch and the femoral site of measurement. The SphygmoCor values were multiplied by 1.103 due to the fact that the subtracted carotid–femoral distance might underestimate the real distance travelled by 10.3% [16, 17].

#### 2.3.4 Carotid Echo‐Tracking

Carotid artery distensibility was measured 1.5 cm from the bifurcation using an Aloka Pro‐Sound alpha 10 ultrasound machine, recording parameters such as elasticity (Ep), β stiffness coefficient, arterial compliance, local AIx, and local PWV.

#### 2.3.5 Echocardiography

Left ventricular mass index (LVMI) was calculated using the Dubois formula, with LVH defined as LVMI ≥ 125 g/m^2^ in men and ≥ 110 g/m^2^ in women [18].

#### 2.3.6 Carotid Intima‐Media Thickness (IMT) and Plaques

IMT was measured in the common carotid artery (1.5 cm) from the bifurcation using a high‐frequency linear probe. Three manual measurements were performed (anterolateral, mediolateral, and posterolateral). Plaques were recorded based on location, thickness, and base.

#### 2.3.7 Urinary Albumin Excretion (UAE)

In most patients, the UAE was determined using the albumin to creatinine ratio (ACR) in the first‐morning urine. Only in those with macroalbuminuria was the 24‐h urine measured by immunonephelometry. Microalbuminuria was defined as a UAE of 30–300 mg/24 h or an ACR greater than 30 mg/g creatinine, and macroalbuminuria as a UAE of 300–500 mg/24 h or an ACR greater than 300 mg/g creatinine.

#### 2.3.8 Glomerular Filtration Rate (GFR)

Estimated GFR (eGFR) was calculated using the CKD‐EPI formula, with CKD classified into stages based on eGFR [19].

#### 2.3.9 CV Events

CV complications were tracked from the first visit until May 30, 2018, using peer‐reviewed analysis of electronic health records. CV complications included hospitalizations for atrial fibrillation (AF), heart failure (HF), stroke, ischemic heart disease (IHD), peripheral artery disease (PAD), or CV death. The first CV event, including its date, was recorded, and AF was confirmed through the EKG.

### 2.4 Statistical Analysis

Quantitative variables are expressed as means and standard deviations (SD), and qualitative variables are expressed as absolute numbers and percentages. Variables with a skewed distribution were expressed as medians (interquartile range). In most statistical analyses, moderate and extreme outliers were excluded by using the interquartile range method.

Considering the active search results for subclinical HMOD, we elaborated on the SCORE of organ damage. We considered LVH (≥ 125 g/m in men and 110 g/m in women), IMT ≥ 0.9 mm, UAE (≥ 30 mg/24 h or albumin‐creatinine ratio ≥ 30 mg/g creatinine), and the presence of atheromatous plaques in any of the right or left carotid territories. In addition to the presence of subclinical HMOD, which was considered dichotomously (presence or absence), the magnitude of the damage was also considered. For this, the measurements of LVH, UAE, IMT, and plaque extension towards the lumen of the artery were divided into quintiles. The final SCORE ranged from 0 to a maximum of 24 points (each marker is initially scored dichotomously [1 point if present, 0 points if absent] and then further graded according to its severity, divided into quintiles [up to 5 additional points]; thus, each marker can contribute up to 6 points, leading to a maximum total of 24 points [4 markers × 6 points each]), and the population was subsequently divided into SCORE deciles. The average values of HTN‐mediated organ damage according to the SCORE categories are shown in Table S1. The relationships between AS parameters and SCORE for subclinical HMOD were established using Pearson or Spearman correlation coefficients. In our study, patients who did not undergo a complete HMOD assessment due to normal BP values were systematically assigned to the lowest SCORE category. Recognizing the potential clustering of individuals with minimal or undetected organ damage, we made a deliberate methodological decision to exclude categories with a SCORE < 1 from the primary analyses. This approach helps avoid underestimation of organ damage that could arise from incomplete evaluations, thereby providing a more accurate representation of the true distribution of subclinical HMOD.

The adjusted survival curves from a Cox proportional hazards model based on the Cox proportional hazards adjusted by age and SBP according to tertiles of the 1st, 2nd, and the delta of cfPWV were calculated to determine the influence of cfPWV on the development of CV events, and Harrell's C statistic was calculated. The *t*‐test for independent samples was used to compare baseline and follow‐up PWV and delta between those with and without CV events. Receiver operating curves (ROC) were used to assess the predictive capacity of the different measures of PWV on CV events. All statistical analyses were performed using the Stata IC v14 software (Stata Corp, 4905 Lakeway Drive, College Station, Texas, 77845, EE.UU.).

### 2.5 Ethical Consideration

All patients signed an informed consent form accepting the use of their clinical data for this study. The project was approved by the Research Institute of the Clinical Hospital of Valencia (INCLIVA). The study was carried out in accordance with the guidelines of good clinical practice, the criteria for medical and biomedical research of the Declaration of Helsinki [20], and the Organic Law 3/2018 on data protection.

## 3 Results

The final sample includes 1031 patients (mean age 52.4 and 40.5% women; mean BMI, 31.3 kg/m^2^; 80% hypertensives) who underwent partial or complete CV phenotyping. All patients had at least one measurement of vascular stiffness parameters, and there was a subgroup of 174 patients with at least another second measurement during follow‐up. The main characteristics of the selected population according to the presence or absence of HTN and the number of cfPWV determinations (one or two) are shown in Table 1.

**TABLE 1:** Main characteristics of the study population according to the hypertension status and number of PWV determinations.

Variables	Hypertensives (*N* = 831)	Normotensives (*N* = 200)	One cfPWV measurements (*N* = 857)	Two cfPWV measurements (*N* = 174)
Age (years)	54.8 ± 14.8	40.9 ± 12.0 ^***^	52.13 ± 0.49	52.37 ± 0.81
Female (*N*%)	480 (42.3)	267 (64.34)^***^	365 (42.6)	53 (30.5)^**^
BMI (kg/m^2^)	29.1 ± 4.4	25.4 ± 4.5^***^	28.4 ± 4.8	28.2 ± 4.1
Brachial SBP (mmHg)	136.5 ± 18.5	123.5 ± 15.1^***^	133.6 ± 19.6	135.7 ± 15.6
Brachial DBP (mmHg)	86.1 ± 11.7	76.2 ± 10.8^***^	84.2 ± 12.4	83.8 ± 11.5
Brachial PP (mmHg)	50.5 ± 15.5	47.3 ± 11.7^**^	49.5 ± 15.1	52.4 ± 13.7^*^
HR (bpm)	69.6 ± 12.4	69.9 ± 12.2	69.3 ± 12.1	72.0 ± 13.6^**^
Central SBP (mmHg)	126.4 ± 17.9	111.9 ± 14.4^***^	123.2 ± 18.6	125.1 ± 15.7
Central DBP (mmHg)	87.1 ± 12.2	77.3 ± 10.9^***^	85.1 ± 12.7	84.9 ± 11.4
Central PP (mmHg)	39.5 ± 13.5	35.2 ± 10.5^***^	38.3 ± 13.5	40.2 ± 10.1
24 h SBP (mmHg)	131.0 ± 13.1	124.6 ± 7.9^***^	129.7 ± 12.6	130.2 ± 12.3
24 h DBP (mmHg)	81.2 ± 8.8	74.7 ± 11.5^***^	79.8 ± 10.0	80.3 ± 8.6
24 h MAP (mmHg)	100.0 ± 9.6	95.3 ± 73^***^	99.5 ± 9.6	97.9 ± 8.3
24 h HR (bpm)	72.3 ± 9.5	69.2 ± 9.5^*^	71.3 ± 9.5	72.9 ± 9.7
Day SBP (mmHg)	134.8 ± 13.7	128.3 ± 8.2^***^	133.4 ± 13.4	134.7 ± 11.8
Day DBP (mmHg)	84.5 ± 9.5	79.5 ± 7.6^***^	83.5 ± 9.5	84.0 ± 9.0
Day MAP (mmHg)	103.3 ± 10.3	98.9 ± 7.5^**^	102.7 ± 10.2	101.3 ± 9.7
Day HR (lpm)	74.9 ± 10.4	72.8 ± 10.7	74.2 ± 10.5	75.6 ± 10.4
Night SBP (mmHg)	122.7 ± 14.1	115.2 ± 9.0^***^	121.0 ± 13.6	122.6 ± 13.5
Night DBP (mmHg)	73.6 ± 9.4	67.3 ± 6.5^***^	72.3 ± 9.3	72.8 ± 9.3
Night MAP (mmHg)	92.4 ± 10.4	85.6 ± 9.3^***^	91.3 ± 10.7	90.4 ± 9.8
Night HR (bpm)	65.4 ± 8.9	60.4 ± 8.6^***^	63.9 ± 8.9	66.5 ± 9.3^*^
c‐f PWV (m/s)	8.8 ± 2.1	6.9 ± 1.5^***^	8.61 ± 2.3	8.58 ± 2.1
AIx (%)	27.5 ± 11.9	22.8 ± 13.7^***^	26.5 ± 12.7	28.3 ± 10.7^*^
AIx corrected by HR (%)	24.7 ± 10.8	20.1 ± 13.9^***^	23.4 ± 11.8	26.9 ± 11.1^**^
Augmentation (mmHg)	11.5 ± 7.4	8.2 ± 5.7^***^	10.67 ± 7.2	12.07 ± 6.7^*^
Carotid beta parameter^¶^	8.2 ± 3.7	6.5 ± 2.5^***^	7.78 ± 3.41	9.44 ± 5.3^**^
Carotid Ep module (kPa)^¶^	121.1 ± 61.3	83.5 ± 36.5^***^	111.8 ± 55.3	142.3 ± 103.2^**^
Carotid arterial compliance (mm^2^/kPa)^¶^	0.88 ± 0.35	1.6 ± 7.1^*^	1.04 ± 3.31	0.80 ± 0.35
Carotid AIx (%)^¶^	15.6 ± 12.4	9.3 ± 14.2^***^	14.3 ± 13.2	14.5 ± 11.5
Local carotid PWV (m/s)^¶^	6.7 ± 2.6	5.6 ± 1.2^***^	6.44 ± 2.4	6.97 ± 2.0
Type 2 diabetes (*N*%)	160 (19.25)	6 (3)^***^	135 (15.7)	31 (17.8)
Dyslipidemia (*N*%)	621 (74.7)	78 (39.0)	563 (65.7)	136 (78.2)^**^
Tobacco (*N*%):				
Past smoker	257 (32.6)	43 (24.2)	249 (31.1)	51 (30.4)
Current smokers	211 (26.8)	40 (22.4)	197 (24.7)	54 (32.2)
Antidiabetic treatments (*N*%)	119 (14.3)	6 (3)^***^	104 (12.1)	21 (12.1)
Hypolipemic treatments (*N*%)	266 (32.1)	20 (10)^***^	219 (25.6)	67 (38.5)^**^
Antihypertensive treatments (*N*%)	572 (69.1)	0 (0)^***^	457 (53.5)	115 (66.1)^**^
LVH (*N*%)	166 (20.1)	5 (2.5)	138 (16.1)	33 (18.9)
Carotid atherosclerosis (*N*%)	204 (24.6)	14 (7)^***^	190 (22.2)	28 (16.1)
Previous cardiovascular events (*N*%)	16 (1.9)	0 (0)^*^	11 (1.3)	5 (2.9)
Glucose (mg/dL)	106.0 ± 25.3	95.0 ± 17.1^***^	103.8 ± 23.3	105.6 ± 29.6
Uric acid (mg/dL)	5.95 ± 1.4	5.53 ± 5.1^*^	5.9 ± 2.6	5.7 ± 1.4
Estimated GFR by CKD‐EPI formula (mL/min)	71.8 ± 16.5	76.0 ± 7.5	92.2 ± 19.3	90.8 ± 16.0
Total cholesterol (mg/dL)	205.3 ± 37.5	199.3 ± 49.2^*^	203.5 ± 39.2	207.6 ± 35.9
LDL cholesterol (mg/dL)	131.9 ± 31.6	127.8 ± 35.5^*^	131.3 ± 32.1	130.8 ± 33.7
HDL cholesterol (mg/dL)	52.0 ± 12.7	56.4 ± 13.4^***^	52.7 ± 13.0	53.2 ± 12.8
Triglycerides (mg/dL)	140.8 ± 88.5	108.9 ± 67.7^***^	135.4 ± 89.4	133.4 ± 68.5
HbA1C (%)	5.9 ± 0.9	5.7 ± 1.0^*^	6.1 ± 2.3	5.8 ± 1.1
UAE (mg/g of creatinine)	28.0 ± 62.6	15.2 ± 46.2^*^	27.3 ± 62.9	24.7 ± 52.9
IMT (mm)	0.64 ± 0.1	0.52 ± 0.1^***^	0.63 ± 0.16	0.62 ± 0.15
LVM (g/m^2^)	123.7 ± 30.7	103.7 ± 27.2^***^	120.4 ± 31.3	124.7 ± 28.2
Carotid plaques (*N*%)	183 (22.5)	19 (9.8)^***^	259 (31.1)	19 (11)^***^

*Note*: Values are expressed as absolute number and percentage or as mean and standard deviation.

Abbreviations: AIx, Augmentation Index; BMI, body mass index; CVD, cardiovascular disease; DBP, diastolic blood pressure; DM2, diabetes mellitus type 2; eGFR, estimated glomerular filtration rate; h, hours; HBA1C, glycosylated hemoglobin; HDL, high density lipoprotein; HR, heart rate; HTN, arterial hypertension; IMT, intima‐media thickness; LDL, low density lipoprotein; LVH, left ventricular hypertrophy; MAP, mean arterial pressure; PP, pressure pulse; SBP, systolic blood pressure; UAE, urinary albumin excretion; VOP, pulse wave velocity.

^¶^ Values refer to the mean of 6 determinations, 3 for each carotid, anterolateral, mediolateral, and posterolateral.

* Statistically significant differences (*p* value < 0.05);

** Very significant differences (*p* value < 0.01);

*** Highly significant differences (*p* value < 0.001).

As expected, the central and peripheral BP measurements were significantly higher in the hypertensive group than in the normotensive group (*p* < 0.0001), as well as the central and peripheral pulse pressure (PP). cfPWV measurement was significantly higher in the hypertensive group (*p* = 0.0243) than in the normotensive group. AIx corrected by HR was also higher in hypertensive patients, but this difference was not statistically significant. All stiffness parameters obtained by echo‐tracking, including carotid AIx and local carotid PWV, were significantly higher in hypertensive patients than in normotensive patients.

Regarding the characteristics according to the number of cfPWV determinations, it seems that the group with at least one repeated measurement had a higher vascular risk, a higher frequency of male sex, and more CV risk factors. Additionally, the number of patients receiving antihypertensive therapy or lipid‐lowering agents was significantly higher (66.1% and 38.5% vs. 53.5% and 25.6%, *p* < 0.01, for antihypertensive and lipid‐lowering drugs in both groups, respectively). On the other hand, the group with only the baseline cfPWV had more carotid plaques than those with at least one follow‐up cfPWV measurement, but there were no differences in BP levels, either in the office or in ABPM.

In PWA, significantly higher values were found in the group with two determinations, especially for the AIx value corrected for HR, which had a mean of 26.9% compared with 23.4% in the one determination group. Higher values were also found in the two stiffness measurements by echo‐tracking in the group of repeated determinations, carotid Ep module, and carotid beta parameter, but not in AIx or local carotid PWV.

### 3.1 Correlation of cfPWV with Clinical, AS Parameters, and Subclinical Organ Damage SCORE

Age emerged as a key determinant of AS, with a strong correlation to cfPWV in both men and women (Figure S1). A critical threshold of 10 m/s suggested increased vascular damage risk, typically observed around age 60.

Central BP demonstrated more robust correlations with AS parameters compared to peripheral measurements. The most significant relationship was between central SBP and augmentation pressure (Table S2).

**FIGURE 1:**
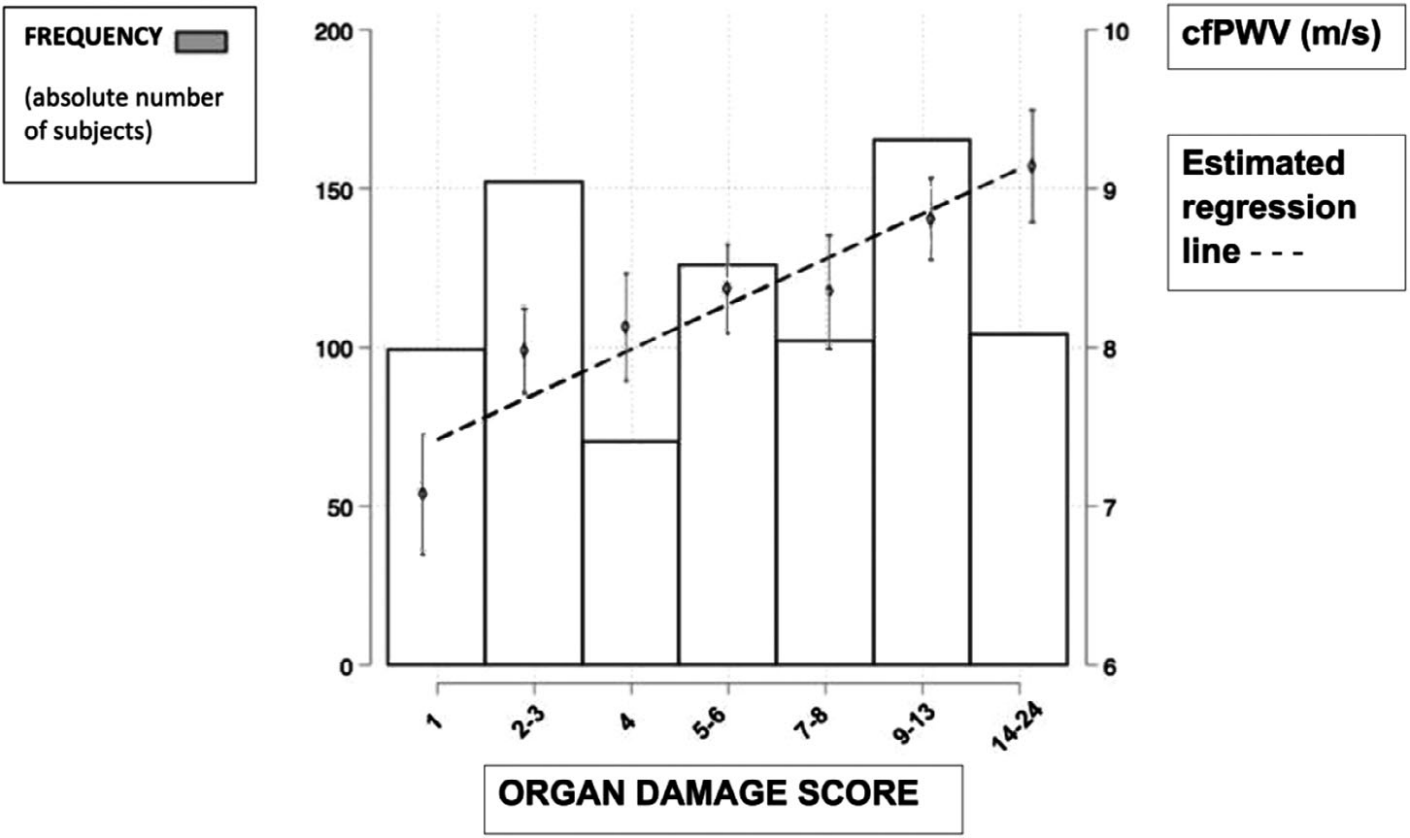
Average values of the cfPWV for each category of the hypertension mediated organ damage (HMOD) score with the estimated linear regression line between the PWV and SCORE. The figure also shows the histogram with the absolute number of individuals in each category of the score.

Our novel HMOD score showed a notable positive correlation with cfPWV (*r* = 0.194), revealing a progressive relationship between AS and organ damage (Figure 1 and Table S2). The carotid Ep module stood out with the strongest correlations, while local stiffness measurements showed more modest interrelationships. Notably, the correlation between cfPWV and local carotid PWV remained relatively weak (*r* = 0.28), as detailed in the correlation matrix in Table S2. Despite no significant correlation between SCORE and cfPWV delta, a trend emerged suggesting increased delta across SCORE categories (Figure S2).

### 3.2 Changes in the cfPWV and Development of CV Events

In our database, 174 patients had at least one repeated cfPWV with an average follow‐up of 2.4 years. There were only 12 CV events as follows: 5 cases of AF, 5 hospitalizations for stroke, one hospitalization due to PAD, and 1 CV death, with the average follow‐up of 3.8 years from the second cfPWV determination.

**FIGURE 2:**
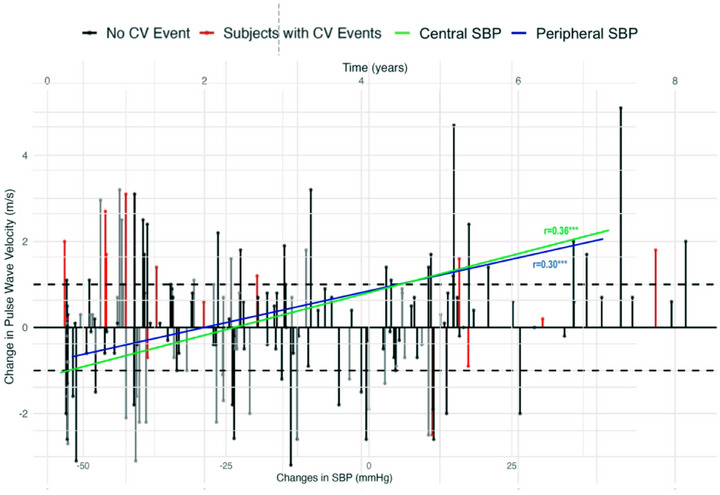
Changes in cfPWV during the follow‐up for patients with and without cardiovascular complications. Estimated regression line between changes in cfPWV and changes in central and peripheral SBP.

Despite the high variability in cfPWV evolution among the 174 subjects with two measurements, the majority of those who experienced CV events during follow‐up showed a significant increase in cfPWV greater than 1 m/s (Figure 2). The strongest correlation between changes in cfPWV over time and BP was observed with changes in central SBP, followed by changes in peripheral SBP (Figure 2). Additionally, there were significant positive correlations with the second measurement of both central and peripheral SBP, but not with the first measurement.

**FIGURE 3:**
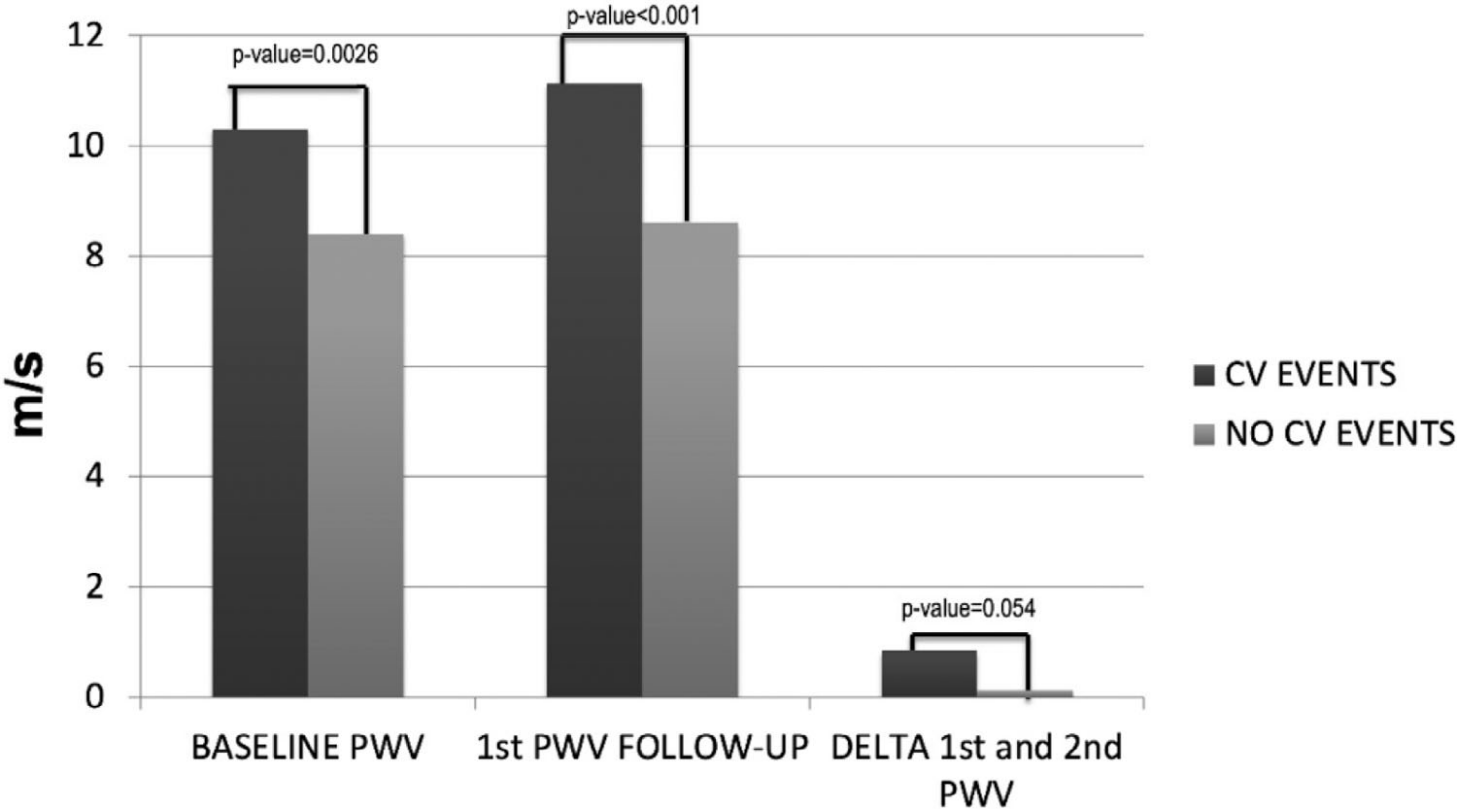
Bar graph for the comparison of the baseline, the follow‐up cfPWV, and the delta of increase in patients with or without cardiovascular events.

Both the baseline and follow‐up cfPWV and the delta of increase were greater in patients with events (Figure 3). The only exception was a 65‐year‐old male, a former smoker with untreated HTN and dyslipidemia, who was diagnosed with AF. His baseline cfPWV was 10.8 m/s and had a delta of −2.5 m/s during the follow‐up.

Although the second PWV determination was better at discriminating events than the first or the delta value, these differences were not statistically significant (ROC area 0.79 vs. 0.69 and 0.66, respectively). The value with the best discrimination for vascular events was for the second cfPWV measurement, with a cutoff point of 9.10 m/s (Figure S3, top).

The survival curves for tertiles of the 1st, 2nd, and delta PWV further demonstrated the relevance of each cfPWV measurement in the development of CV complications. As with the ROC curves, the best predictive capacity was for the 2nd cfPWV, followed by the delta and the 1st cfPWC (0.86 vs. 0.82 vs. 0.80 for Harrell's *C* statistic) (Figure S3, bottom).

## 4 Discussion

Despite its limitations, our study highlights the potential usefulness of cfPWV in clinical practice as an integrated marker of CV risk not only in hypertensives but also in the general population with other CV risk factors. The key findings were as follows: (1) cfPWV serves as a simple marker for HMOD at different levels; (2) cfPWV is significantly related to other stiffness variables; (3) a 1 m/s or higher increment during follow‐up could predict CV events; (4) the optimal threshold for aortic subclinical organ damage associated with CV events may be lower than the guideline recommendations.

### 4.1 First Finding

We found a significant association between cfPWV and HMOD at other levels, which may help explain the predictive value of cfPWV changes for CV events. Our study did not find a significant correlation between SCORE and the delta of cfPWV, but there was a trend toward greater differences in cfPWV measurements as SCORE increased, similar to those who developed CVE. Our findings also revealed a clear association between cfPWV and the presence of subclinical organ damage at various levels, as demonstrated in previously published studies [21, 22, 23].

Several studies have shown that central arterial pressure is more strongly associated with organ damage and CV events than brachial pressure [24, 25, 26]. Our data also showed a better correlation between SCORE and central BP, particularly central DBP, and to some extent with peripheral DBP. Unlike SBP, where amplification occurs from the aorta to the brachial artery, DBP shows minimal variation between the central and brachial levels, often slightly higher centrally. Given the mean age of our population (52.5 years), DBP may relate to organ damage as much as SBP. Recent evidence indicates that both SBP and DBP can predict CV events [27].

### 4.2 Second Finding

cfPWV can be supplemented by other stiffness parameters. Significant correlations were found between different AS measurements, particularly between the AIx values determined by tonometry and carotid echo tracking. This high correlation has been described by other authors and may be influenced by antihypertensive medications [28]. cfPWV was significantly correlated with local carotid PWV, although the correlation was weaker than that reported in other studies [29]. As other studies show [30], our results support using AIx and AS parameters obtained by echo‐tracking as complementary measures to cfPWV. Although all these parameters can provide complementary information, how to use these measures in combination in routine clinical practice has yet to be determined.

### 4.3 Third Finding

Many studies [31, 32, 33, 34] support the prognostic value of cfPWV in predicting CV events and mortality; however, data on the predictive utility of changes in cfPWV are limited. The observed variability in cfPWV changes over time suggests that AS progression is an important CV risk factor. Our study found that patients who experienced CV events during follow‐up showed a significant increase of > 1 m/s in cfPWV. While the incremental predictive value of cfPWV beyond SBP and age appears modest, the potential clinical significance of cfPWV changes warrants further investigation [35, 36, 37, 38]. Our HMOD score demonstrated a lower predictive value for CV events compared to cfPWV, with an area under the curve (AUC) of 0.557 and a Harrell's *C* statistic, adjusted for age and SBP, of 0.79.

Some critics argue that the high dependence of cfPWV on age and BP limits its predictive ability for CV outcomes [39, 40]. Age is a critical nonmodifiable factor for interpreting cfPWV values and their evolution. AS increases progressively with age and is likely accelerated by cumulative aortic wall damage and increasing prevalence of risk factors [41]. Other factors, such as sex, also influence cfPWV, with increases being slightly lower in women than in men, as observed by Al Ghatrif et al. [42]. In our study, the threshold of 10 m/s was crossed at age 60 years (later for women), increasing the likelihood of vascular organ damage and CV risk.

Like other studies, ours shows a significant correlation between AS and BP, especially central BP over brachial BP [42, 43, 44]. The correlations between stiffness parameters and DBP were generally approximately half those for SBP, both centrally and peripherally. Similar results were found in the LIFE‐Adult study, a large population‐based cohort [45], and a recent study showed a higher correlation between PWV and SBP than DBP [46].

### 4.4 Fourth Finding

The threshold for arterial damage measured by cfPWV might be lower than the 10 m/s suggested by guidelines [47, 48, 49, 50]. This is supported by studies in hemodialysis patients, which found that the second cfPWV measurement better predicted CV events [48]. Recent evidence suggests that the threshold for CV events related to PWV is approximately 9 m/s [51].

### 4.5 Strengths and Limitations

Our retrospective study has several limitations. The group with repeated measurements was biased, including patients with a higher vascular risk. As patients belong to a cardiovascular risk unit, this may not reflect the general population. Additionally, the small number of registered CV events due to the short follow‐up period in a relatively young population, primarily in primary prevention, limits the study's power. Another bias was that not all variables included in the SCORE were measured in all subjects, leading to the exclusion of those with very low scores from the analysis of the relationship between cfPWV and HMOD. To avoid false positives, given that minor angulations in M‐mode echocardiography can lead to overestimation of left ventricular mass, we opted for the higher LVH thresholds (125 g/m^2^ for men and 110 g/m^2^ for women) instead of the more commonly used cut‐offs (115 and 95 g/m^2^, respectively). Moreover, the low number of CV events limited the power to assess the impact of cfPWV changes using the Cox proportional hazards model, leading to the inclusion of AF as an event, although AF could have been paroxysmal or asymptomatic before its diagnosis. As it was shown in Figure 2, we observed instances in which cfPWV rose despite a notable decrease in SBP, suggesting that factors beyond BP—such as AS progression, measurement variability, and individual patient characteristics—may affect changes in cfPWV over time; indeed, the overall correlation between SBP increases and cfPWV increases was relatively weak.

Despite these limitations, the strengths of this study include the analysis of the relationship between different AS measurements and subclinical organ damage in a large sample of patients with follow‐up measurements in a real clinical setting. This is important for evaluating the effect of treatment on these parameters and their potential as surrogate markers. Although the sample size with follow‐up measures of cfPWV was small, the observed trends remained significant. Another strength is that our risk SCORE considers not only the presence or absence of organ damage, but also its extent.

## 5 Conclusions

Our findings suggest that cfPWV is a simple and effective tool for detecting the burden of HMOD and assessing CV risk, as well as identifying vascular stiffness in other territories. Although it was not our primary objective and our analysis was limited by the sample size and number of events, a second cfPWV measurement during regular follow‐up may help to better identify patients at risk of CV complications—particularly those showing an increase in PWV greater than 1 m/s. Moreover, as often occurs with continuous variables, the threshold associated with increased CV risk appears to be lower than the commonly accepted 10 m/s threshold for aortic HMOD.

### 5.1 Future Prospects

Monitoring cfPWV over time, given its correlation with the extent of HMOD, could potentially improve HTN management by guiding treatment decisions and identifying patients at higher CV risk, particularly those demonstrating progressive increases in cfPWV. Future studies are needed to establish optimal assessment intervals and clinical thresholds for integrating cfPWV into routine clinical practice.

## Author Contributions

Conceptualization: Fernando Martínez Garcia, Josep Redon, Oscar Calaforra, and Grethzel Prado. Methodology: Oscar Calaforra, Fernando Martínez Garcia, María J. Forner, Sara Vela, Carlos Bea, Gernot Pichler, Ana de Gracia, and Lucas Serna. Formal Analysis: Grethzel Prado, Gernot Pichler, Oscar Calaforra, and Fernando Martínez Garcia. Data curation: Grethzel Prado, María J. Forner, Sara Vela, Carlos Bea, Gernot Pichler, Ana de Gracia, and Lucas Serna. Writing–original draft: Grethzel Prado and Oscar Calaforra. Writing–review and editing: Fernando Martínez Garcia, Josep Redon, Enrique Rodilla, Sara Vela, Carlos Bea, Gernot Pichler, and María J. Forner. All authors contributed to the conception, drafting, and final approval of the manuscript.

## Ethics Statement

This study involved human participants and was approved by the local ethics committee of the Research Institute of the Clinical Hospital of Valencia (INCLIVA). All procedures were conducted in accordance with the ethical standards outlined in the Declaration of Helsinki.

## Consent

Informed consent was obtained from all participants included in this study.

## Conflicts of Interest

The authors declare no conflicts of interest.

## Supporting information



Supporting information

Supporting information

Supporting information

Supporting information

Supporting information

## Data Availability

The data supporting this study are available from the corresponding author upon reasonable request. No material from other sources has been reproduced in this manuscript. This study is not a clinical trial and does not require registration.
