# Supplementary material for: Usefulness of Arterial Stiffness as an Integrated Marker of Cardiovascular Risk
Source: J Clin Hypertens (Greenwich). 2025 Mar 24;27(3):e70038. doi: 10.1111/jch.70038 (PMC11932553; doi:10.1111/jch.70038)
Supplement: Supplementary file 1 — Supporting information [file JCH-27-e70038-s002.docx]

***Supplementary Table 1.*** Mean values or hypertension-mediated organ damage according to the SCORE.

| SCORE  CATEGORIES | score  values | MALB  % | lvh  % | imt  % | IMT | right side plaque size  mm | left side plaque size  mm | lvm  g/m^2^ | uae  mg/g |
| --- | --- | --- | --- | --- | --- | --- | --- | --- | --- |
| 1 |  |  |  |  |  |  |  |  |  |
| 2 |  |  |  |  |  |  |  |  |  |
| 3 |  |  |  |  |  |  |  |  |  |
| 4 | 1 |  | 6.1 |  | 0.44 ± 0.03 |  |  | 81.2 ± 8.1 | 1.84 ± 0.83 |
| 5 | 2-3 |  | 2.0 |  | 0.53 ± 0.07 | 1.47 ± 0.16 |  | 93.8 ± 14.2 | 4.67 ± 1.67 |
| 6 | 4 |  | 3.0 |  | 0.58 ± 0.08 |  | 1.38 ± 0.17 | 103.6 ± 15.6 | 10.46 ± 6.35 |
| 7 | 5-6 | 35.6 | 8.5 | 5.8 | 0.61 ± 0.12 | 1.61 ± 0.36 | 1.33 ± 0.19 | 106.2 ± 24.3 | 51.73 ± 115.50 |
| 8 | 7-8 | 15.9 | 15.2 | 13.5 | 0.61 ± 0.12 | 1.67 ± 0.36 | 1.50 ± 0.39 | 112.3 ± 23.1 | 27.82 ± 71.49 |
| 9 | 9-13 | 22.1 | 33.1 | 29.4 | 0.68 ± 0.14 | 3.09 ± 11.17 | 1.88 ± 0.52 | 130.2 ± 33.1 | 32.62 ± 64.60 |
| 10 | 14-24 | 31.6 | 55.8 | 59.4 | 0.80 ± 0.19 | 2.24 ± 0.79 | 2.20 ± 0.67 | 137.0 ± 28.9 | 46.54 ± 78.2 |

*Values are expressed as percentages or mean ± standard deviation.
