# Supplementary material for: Usefulness of Arterial Stiffness as an Integrated Marker of Cardiovascular Risk
Source: J Clin Hypertens (Greenwich). 2025 Mar 24;27(3):e70038. doi: 10.1111/jch.70038 (PMC11932553; doi:10.1111/jch.70038)
Supplement: Supplementary file 2 — Supporting information [file JCH-27-e70038-s004.docx]

**Supplementary Table 2**. Matrix of correlations between arterial stiffness parameters themselves and with blood pressure.

|  | **SBP** | **DBP** | **PP** | **cSBP** | **cDBP** | **Augmentation** | **AIx** | **AIx75** | **Beta Coeficiente** | **Ep**  **module** | **Carotid**  **compliance** | **AIx carotídeo** | **Local cPWV** | **cfPWV** | **SCORE** |
| --- | --- | --- | --- | --- | --- | --- | --- | --- | --- | --- | --- | --- | --- | --- | --- |
| **SBP** | X |  |  |  |  |  |  |  |  |  |  |  |  |  |  |
| **DBP** | 0.6114* | x |  |  |  |  |  |  |  |  |  |  |  |  |  |
| **PP** | 0.7696* | -0.0053 | X |  |  |  |  |  |  |  |  |  |  |  |  |
| **cSBP** | 0.9569* | 0.7018* | 0.6554* | x |  |  |  |  |  |  |  |  |  |  |  |
| **cDBP** | 0.6365* | 0.9865* | 0.0244 | 0.7011* | x |  |  |  |  |  |  |  |  |  |  |
| **Augmentation** | 0.4968* | 0.1222* | 0.5268* | 0.6491* | 0.1314* | x |  |  |  |  |  |  |  |  |  |
| **AIx** | 0.1148* | 0.1252* | 0.0425 | 0.3570* | 0.1360* | 0.7767* | x |  |  |  |  |  |  |  |  |
| **AIx75** | 0.1635* | 0.2410* | 0.0075 | 0.3682* | 0.2487* | 0.6986* | 0.8810* | x |  |  |  |  |  |  |  |
| **Beta coefficient**  **Beta** | 0.2757* | 0.0348 | 0.3372* | 0.2733* | 0.0399 | 0.2893* | 0.1236 | 0.2095* | x |  |  |  |  |  |  |
| **Ep module** | 0.5004* | 0.2606* | 0.4494* | 0.4956* | 0.2662* | 0.3725* | 0.1441 | 0.2394* | 0.9529* | x |  |  |  |  |  |
| **Carotid**  **compliance**  **arterial** | -0.1087 | -0.1059 | -0.0554 | -0.1016 | -0.1063 | -0.0099 | 0.0307 | 0.0076 | -0.1435 | -0.1380 | x |  |  |  |  |
| **Carotid AIx** | 0.1984* | 0.2587* | 0.0377 | 0.3840* | 0.2548* | 0.6291* | 0.7143* | 0.6512* | 0.1372 | 0.1729* | -0.0634 | x |  |  |  |
| **Local cPWV** | 0.2576* | 0.1611* | 0.2094* | 0.2615* | 0.1623* | 0.2019* | 0.1069 | 0.1795* | 0.5426* | 0.5527* | 0.0296 | 0.1203 | x |  |  |
| **cfPWV** | 0.4277* | 0.1864* | 0.3841* | 0.4507* | 0.2005* | 0.3831* | 0.2242* | 0.3160* | 0.3827* | 0.4524* | -0.0817 | 0.3004* | 0.2835* | x |  |
| **SCORE** | 0.0791 | 0.2053* | -0.0564 | 0.1308* | 0.2141* | -0.0364 | -0.0119 | -0.0032 | 0.2476* | 0.2970* | -0.0422 | 0.1892* | 0.1654* | 0.1944* | x |

*Significant correlation with p-value<0.01 and Bonferroni adjustments. SBP: systolic blood pressure; DBP: diastolic blood pressure; PP: pulse pressure; cSBP: central systolic blood pressure; cDBP: central diastolic blood pressure; AIx augmentation index;AIx75: augmentation index corrected by heart rate at 75 beats for minute; cPWV carotid pulse wave velocity; cfPWV: carotid-femoral pulse wave velocity.
