# Supplementary figures and images for: Usefulness of Arterial Stiffness as an Integrated Marker of Cardiovascular Risk
Source: J Clin Hypertens (Greenwich). 2025 Mar 24;27(3):e70038. doi: 10.1111/jch.70038 (PMC11932553; doi:10.1111/jch.70038)

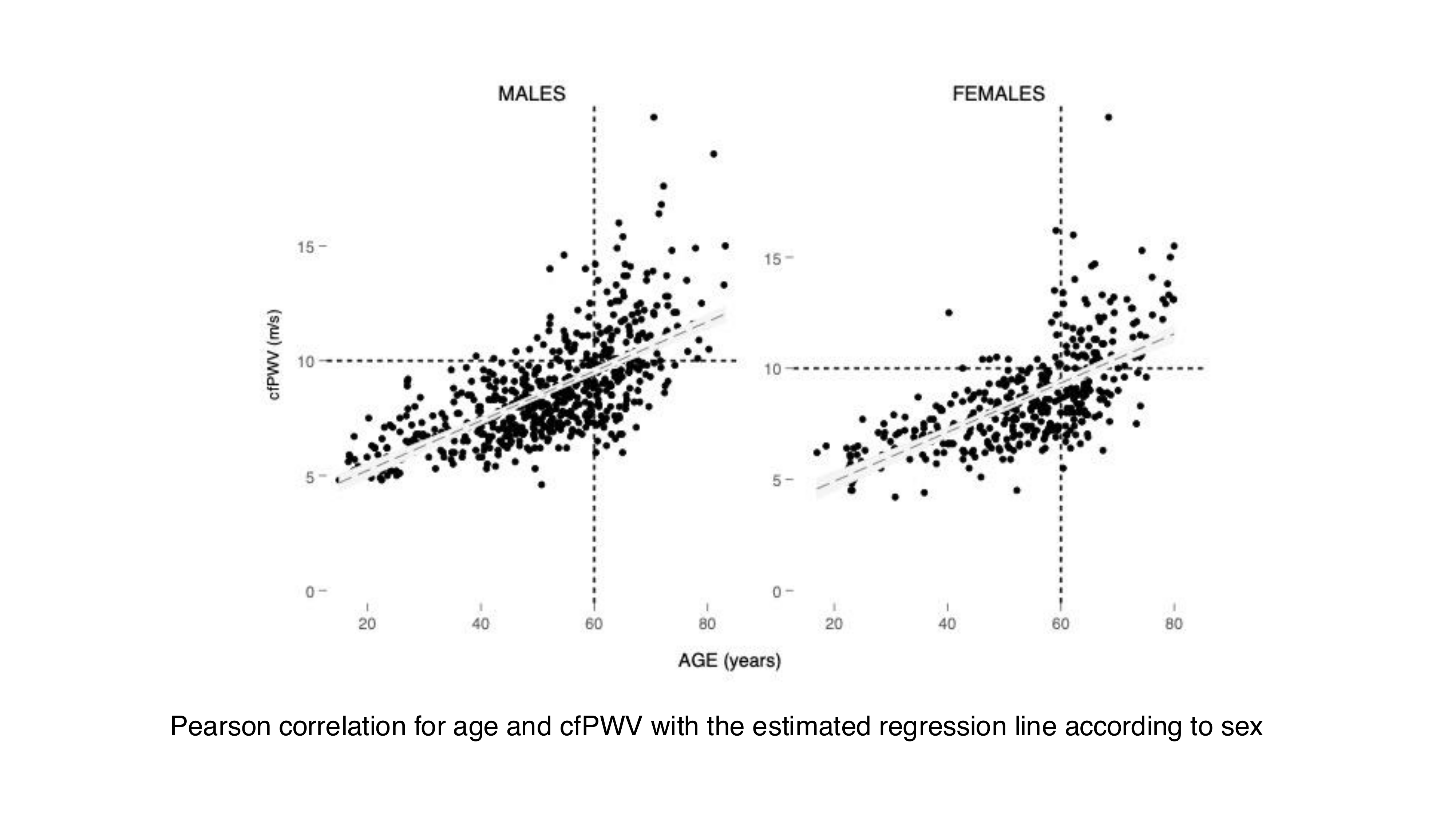

Supplement: Supplementary file 3 — Supporting information [file JCH-27-e70038-s003.tif]

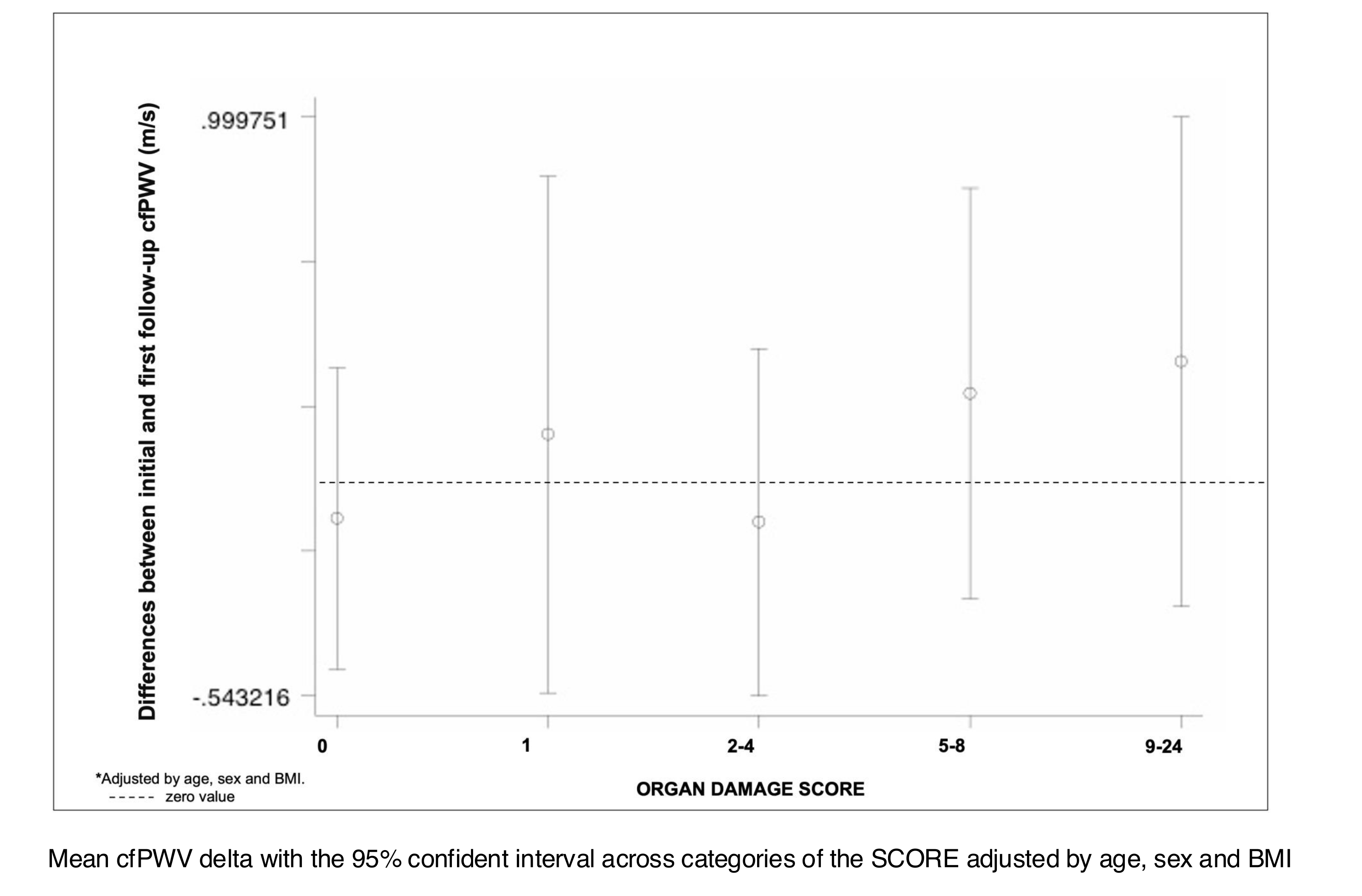

Supplement: Supplementary file 4 — Supporting information [file JCH-27-e70038-s001.tif]

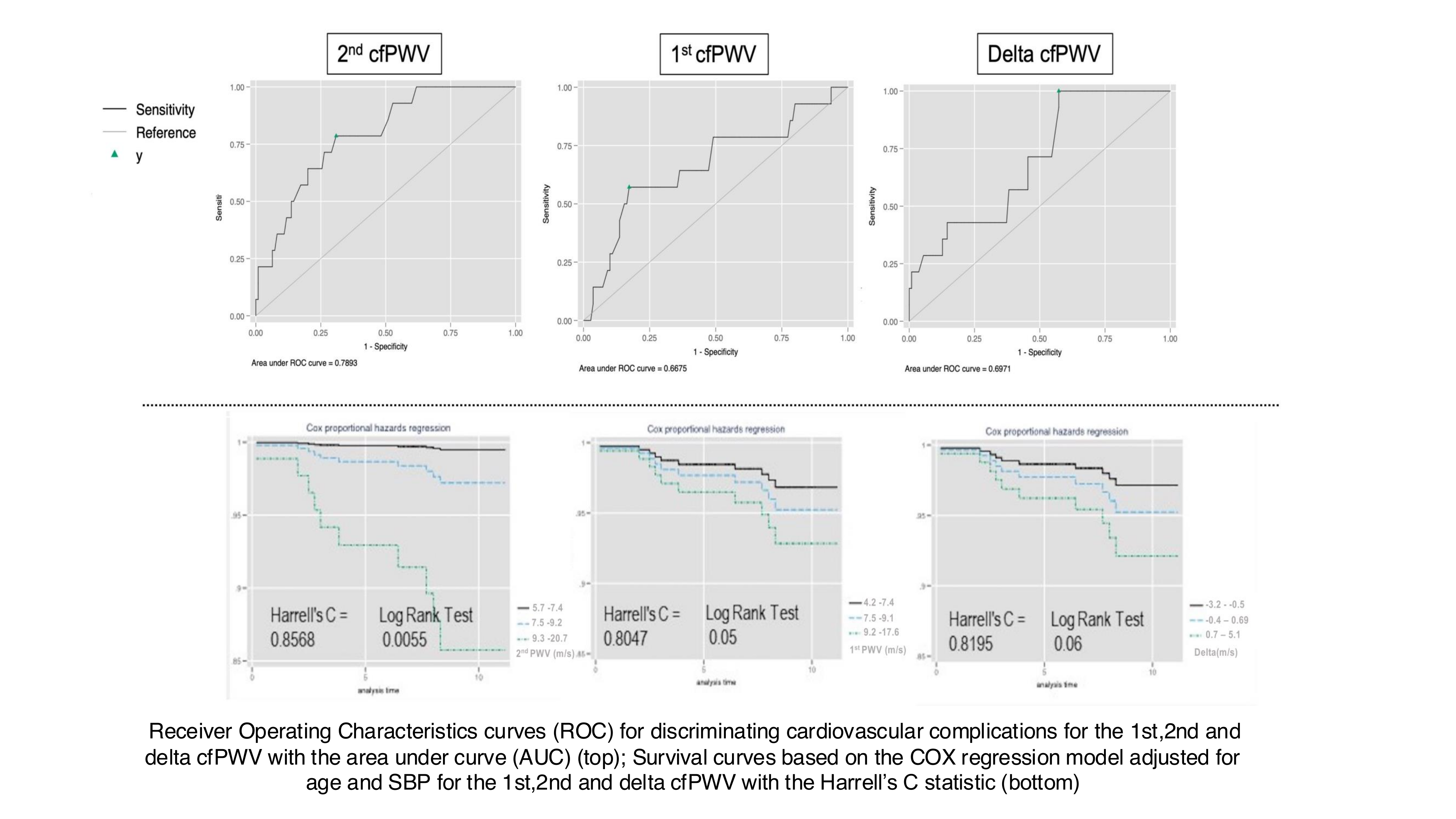

Supplement: Supplementary file 5 — Supporting information [file JCH-27-e70038-s005.tif]
